# Association of Brain-Derived Neurotrophic Factor Gene Val66Met Polymorphism with Primary Dysmenorrhea

**DOI:** 10.1371/journal.pone.0112766

**Published:** 2014-11-10

**Authors:** Lin-Chien Lee, Cheng-Hao Tu, Li-Fen Chen, Horng-Der Shen, Hsiang-Tai Chao, Ming-Wei Lin, Jen-Chuen Hsieh

**Affiliations:** 1 Institute of Brain Science, National Yang-Ming University, Taipei, Taiwan; 2 Department of Physical Medicine and Rehabilitation, Cheng Hsin General Hospital, Taipei, Taiwan; 3 Integrated Brain Research Unit, Division of Clinical Research, Department of Medical Research, Taipei Veterans General Hospital, Taipei, Taiwan; 4 Department of Education and Research, Taipei City Hospital, Taipei, Taiwan; 5 Division of Basic Research, Department of Medical Research, Taipei Veterans General Hospital, Taipei, Taiwan; 6 Department of Obstetrics and Gynecology, Taipei Veterans General Hospital, Taipei, Taiwan; 7 Department of Obstetrics and Gynecology, Faculty of Medicine, School of Medicine, National Yang-Ming University, Taipei, Taiwan; 8 Institute of Public Health, National Yang-Ming University, Taipei, Taiwan; Institut Jacques Monod, France

## Abstract

Primary dysmenorrhea (PDM), the most prevalent menstrual cycle-related problem in women of reproductive age, is associated with negative moods. Whether the menstrual pain and negative moods have a genetic basis remains unknown. Brain-derived neurotrophic factor (BDNF) plays a key role in the production of central sensitization and contributes to chronic pain conditions. BDNF has also been implicated in stress-related mood disorders. We screened and genotyped the *BDNF* Val66Met polymorphism (rs6265) in 99 Taiwanese (Asian) PDMs (20–30 years old) and 101 age-matched healthy female controls. We found that there was a significantly higher frequency of the Met allele of the *BDNF* Val66Met polymorphism in the PDM group. Furthermore, *BDNF* Met/Met homozygosity had a significantly stronger association with PDM compared with Val carrier status. Subsequent behavioral/hormonal assessments of sub-groups (PDMs = 78, controls = 81; eligible for longitudinal multimodal neuroimaging battery studies) revealed that the *BDNF* Met/Met homozygous PDMs exhibited a higher menstrual pain score (sensory dimension) and a more anxious mood than the Val carrier PDMs during the menstrual phase. Although preliminary, our study suggests that the *BDNF* Val66Met polymorphism is associated with PDM in Taiwanese (Asian) people, and *BDNF* Met/Met homozygosity may be associated with an increased risk of PDM. Our data also suggest the *BDNF* Val66Met polymorphism as a possible regulator of menstrual pain and pain-related emotions in PDM. Absence of thermal hypersensitivity may connote an ethnic attribution. The presentation of our findings calls for further genetic and neuroscientific investigations of PDM.

## Introduction

Primary dysmenorrhea (PDM), defined as pain associated with menstruation in the absence of organic disease, affects 90% of adolescent girls and more than 50% of menstruating women worldwide [1]–[4]. The related suffering is described as severe and distressing by 10–20% of the subjects with this condition [2]–[4]. Despite the unclear pathogenesis of PDM, myometrial hypercontractility and vasoconstriction are the two most widely accepted mechanisms [5], [6]. In addition, prostaglandins, chemokines, cytokines, growth factors, oxytocin, leukotrienes, and vasopressin, either within the uterus itself or in menstrual fluid or peripheral blood, are potential contributors to altered uterine physiology [7]–[10]. Furthermore, PDM can be associated with hampered daily function [11], decreased self-rated overall health [12], and negative moods (e.g., depressive and anxious symptoms) [13], [14]. However, to our knowledge there is no report in the literature discussing possible genetic causes of PDM in terms of the pain experience and associated emotions.

Brain-derived neurotrophic factor (BDNF), a member of neurotrophin family, supports neuronal growth, differentiation and survival in the peripheral and central nerve systems, and induces long-lasting changes in synaptic plasticity [15], [16]. BDNF is considered a pain modulator because of its involvement in activity-dependent synaptic plasticity within the pain circuitry. With a pro-nociceptive role, BDNF generates the hyperalgesic responses in inflammatory models of pain [17]. BDNF also plays a key role in the production of central sensitization and contributes to chronic pain conditions [17], [18]. Moreover, BDNF is potentially involved in stress-related mood disorders [19], e.g., major depression. Stress [19] or chronic pain [20] decreases the expression of BDNF in brain structures that control mood and contributes to depression.

Natural fluctuations of circulating sex hormones across the menstrual cycle are supposed to be associated with mood and cognition alterations in women. BDNF serves as a mediator of sex hormone effects in the hippocampus, and interaction between estrogen and hippocampal BDNF may underlie the menstrual cycle-related problems [21], [22]. Estrogen regulates *BDNF* gene expression in the brain of animal models, directly by interaction between estrogen receptor and *BDNF* [23]; and indirectly by GABAergic interneurons modulation [24]. During the estrus phase with low estrogen level, *BDNF* Met/Met homozygous mice exhibited increased anxiety-related behaviors because of decreased BDNF availability and affected BDNF signaling [22]. *BDNF* genotype may therefore alter risk for the development, and could play a role in the diagnosis and treatment of menstrual cycle-related problems, specifically PDM.

The *BDNF* Val66Met polymorphism (rs6265) leads to the substitution of methionine (Met) for valine (Val) at codon 66 of the proBDNF protein. The Met allele leads to a reduced activity-dependent secretion of BDNF from neurons and impaired BDNF signaling [25]. The *BDNF* Val66Met polymorphism (rs6265) might be implicated in both chronic pain conditions and mood disorders. It has been reported that the Met allele carriers, compared to the Val homozygotes, displayed augmented cortical processing of experimental pain in chronic low back pain patients, whilst reduced pain processing without the co-existence of chronic pain [26]. The authors proposed a possible epigenetic modulation that enhances *BDNF* Val66Met polymorphism sensitivity in these chronic pain patients to experimentally induced mildly painful stimuli. Animal models with *BDNF* Met allele exhibited increased anxiety-related behaviors under stress, and this phenomenon implicates an effect of the gene (*BDNF* genotype) × environment (stress) interaction on the mood [25], [27]. Although the findings were controversial, previous human studies of *BDNF* Val66Met polymorphism had reported that only *BDNF* Met/Met homozygosity was associated with heightened anxiety [28], [29] and an increased risk of depression [30].

In this study, we firstly set out to explore whether the *BDNF* Val66Met polymorphism (rs6265) could be associated with a higher risk of PDM among Taiwanese (Asian) people. Secondly, we reasoned that *BDNF* Met/Met homozygous PDMs might exhibit heightened menstrual pain perception and more negative emotions as compared to Val carrier PDMs. The relationships between *BDNF* Val66Met polymorphism genotypes and the pain- and emotion-related clinical manifestations of PDM, and the psychophysical assessments (pain sensitivity to experimentally induced thermal skin pain) were examined.

## Methods

### Participants

The inclusion criteria for the PDM group were the followings: (1) 20–30 year-old Taiwanese (Asian) females; (2) a regular menstrual cycle of approximately 27–32 days; (3) a history of PDM longer than 6 months; (4) cramping pain during the menstrual period in the last 6 months rated higher than 4 on a visual analog scale (0 =  not at all, 10 =  the worst pain imaginable); and (5) right-handedness, as confirmed by the Edinburgh Handedness Inventory. The inclusion criteria for the healthy control females were similar to the PDM group, except that the controls did not experience menstrual pain. The exclusion criteria for all the participants were a history of head injury, pathological pituitary gland disease, organic pelvic disease, psychiatric disorder, immediate plans for pregnancy or a positive pregnancy test, a history of childbirth, and having a metal/pacemaker implant. The participants had not used oral contraceptives within 6 months prior to the study. No analgesics had been taken within 24 hours before the study. All the subjects in the PDM group underwent pelvic ultrasonography to exclude secondary dysmenorrhea caused by organic pelvic diseases, such as endometriosis or adenomyosis. All the participants were clinically examined and diagnosed in the Gynecology clinic by a gynecologist (HTC).

Altogether, 306 PDMs and 199 controls were originally screened between August 31, 2011 and September 30, 2013 as intended for multimodal imaging genetics studies (magnetic resonance imaging and magnetoencephalography). During the screening stage, 207 PDMs and 98 controls were excluded due to one or a combination of the following factors: an irregular menstrual cycle, a prolonged or shortened menstrual cycle, inconsistent pain intensity, pelvic abnormalities by ultrasonography, or being unwilling to complete the entire series of genetic, hormonal, behavioral, and multimodal neuroimaging studies. Eventually, a total of 99 PDMs and 101 healthy controls (matched for age and educational level) were enrolled. All these subjects also participated in multimodal imaging genetics experiments (the data will be reported elsewhere). All the experiments were completed within three menstrual cycles due to difficulties in scheduling the brain scanning within one menstrual cycle. The overall study was conducted in accordance with the Declaration of Helsinki and was approved by the Institutional Review Board of Taipei Veterans General Hospital. The participants provided written informed consent.

### Genotyping

Blood samples for genotyping were obtained during the initial examination. Whole blood collected in 10 mL EDTA tubes was stored in 4°C fridge, and DNA extraction was subsequently performed using the Puregene kit following the manufacturer’s guidelines (Gentra Systems, Minneapolis, MN). Commercial TaqMan single-nucleotide polymorphism assays (Applied Biosystems, Foster City, CA) were used for genotyping. The polymerase chain reaction amplification was conducted in total volume of 1L using the following amplification protocol: 50°C for 2 min, 95°C for 10 min, and 40 cycles of 94°C for 15 sec and 62°C for 1 min. Fluorescence measurements were performed using the ABI HT7900 (Applied Biosystems, Foster City, CA), and allele calling was performed with the SDS 2.2 software package (Applied Biosystems). Genotypes were independently assigned to the subjects by two technicians blinded to the subjects’ information.

### Serum Hormone Measurements

The sera extracted from the blood samples drawn during the menstrual phase (within 1–3 days after the start of menstrual flow) and periovulatory phase (12–16 days after the start of menstrual flow) were stored for batch analysis using commercialized assays (UniCel DxC 800 Synchron Clinical Systems, Beckman Coulter, Inc., Brea, CA). The total serum concentrations were assayed using a chemiluminescence immunoassay technique for the estradiol and progesterone, and a radioimmunoassay technique for the testosterone.

#### Behavioral AssessmentsMenstrual pain experience

The PDMs completed the McGill Pain Questionnaire during the initial examination and again during the menstrual phase to assess the recalled overall and current experiences of menstrual pain, respectively.

#### Personality traits and quality of life

All the participants in the two groups completed the Basic Personality Inventory [31] and the SF-36 (quality of life) [32] during the initial examination to assess personality traits and quality of life, respectively.

#### Assessment of anxiety, depressive mood and pain catastrophizing

To evaluate the psychological status across the menstrual cycle, all the participants in the two groups completed self-reported psychological measurements during the menstrual phase and periovulatory phase. Ovulation was confirmed using a urinary luteinizing hormone test [33]–[35]. The psychological battery included the Spielberger State-Trait Anxiety Inventory [36], the Beck Anxiety Inventory [37], the Beck Depression Inventory [38], and the Pain Catastrophizing Scale [39]. The order of the assessments was balanced across the subjects for the menstrual phase and periovulatory phase. Multimodal neuroimaging studies were conducted during these two phases; the results will be reported elsewhere.

#### Quantitative sensory testing for central sensitization

To examine central sensitization throughout the menstrual cycle, as previously reported in studies on white populations [40]–[44], we performed a thermal quantitative sensory testing (thermal sensation and pain thresholds) according to the established protocol of an ascending limit approach for heat pain and a descending limit approach for cold pain [45]. Heat and cold stimuli (TSA 2001-II, MEDOC, Israel) were administered to the bilateral periumbilical areas (T11-dermatome, referral area of menstrual pain) and forearm extensor areas (C7-dermatome, remote control area) during the menstrual phase and periovulatory phase. The baseline temperature was set at 32°C, and all the thresholds were obtained with a ramped stimulation method (1°C/sec). The perception thresholds for cold and warm were determined first, followed by the pain thresholds for cold and heat. The mean thresholds were calculated by averaging three consecutive measurements. The limits of the temperature range were set between 0 and 50°C.

### Statistical Analysis

SPSS Statistics 20.0 (SPSS Inc., Chicago, IL) was used for all the analyses. The data are presented as the means ± standard deviation. The results were considered significant at *P*<0.05 (two-tailed). We did not apply multiple corrections for all these inventories since they access different sensory, affective, cognitive, and physiological dimensions of pain, and the discrete physical and mental impacts of social life. In the first stage of the analysis, the Hardy-Weinberg equilibrium of the *BDNF* genotype distribution and the relationship between genotype and PDM were examined using the chi-square test. We then conducted the second stage of the analysis by investigating the associations between the *BDNF* Val66Met polymorphism and behavioral/hormonal manifestations. The Val/Val and Val/Met subjects were treated as a single genotype category (*BDNF* Val carriers) and compared with the *BDNF* Met/Met homozygotes in the subsequent analyses. This strategy was corroborated by our findings that there were no essential differences in any of the behavioral/hormonal assessments between the *BDNF* Val/Val and *BDNF* Val/Met subjects in the PDM and control groups (all *P*>0.05). Between-group (PDM vs. control) and between-genotype (Met/Met homozygotes vs. Val carriers) differences in demographic characteristics and McGill Pain Questionnaire scores were examined using a two-sample t-test or the Mann-Whitney U test. A two-way analysis of variance (ANOVA) of the Basic Personality Inventory and SF-36 data was conducted for the main effects of group (PDM vs. control) and *BDNF* genotype (Met/Met homozygotes vs. Val carriers), as well as the interaction between them. For the assessments of behavior, quantitative sensory testing and serum hormone levels during the two phases, a general linear model with a repeated measures design was first applied to examine the possible effects of group (PDM vs. control), genotype (Met/Met homozygotes vs. Val carriers), menstrual cycle (menstrual phase vs. periovulatory phase) and the interactions between these factors. To further determine whether different *BDNF* genotypes were associated with different psychological attributes of state anxiety, trait anxiety, Beck Anxiety Inventory, Beck Depression Inventory, and Pain Catastrophizing Scale scores across the menstrual cycle for the respective PDM and control group, repeated-measures ANOVAs were conducted on each psychological measurement to compare the PDM and control groups.

## Results

### Demographic Characteristics (Table 1)

There were no significant differences between the PDM and control groups in age, age at menarche, years of menstruating or average days of one menstrual cycle (*P*>0.05). The PDM group had been experiencing moderate cyclic menstrual pain (recalled overall McGill Pain Questionnaire-pain rating index: 33.3±12.96) for 2 (1.8±0.82) days per month for approximately 10 (9.2±2.70) years.

**Table 1:** Demographic data of the original genotyping groups and the behavioral/hormonal sub-groups.

	PDM		Control	
	Genotyping	Behavioral/hormonal	Genotyping	Behavioral/hormonal
**Subject number**	99	78	101	81
**Age**	23.3 (2.33)	23.4 (2.41)	23.8 (2.35)	23.9 (2.38)
**Age at menarche**	12.0 (1.27)	12.0 (1.25)	12.2 (1.05)	12.3 (1.08)
**Years of menstruating**	11.3 (2.57)	11.4 (2.66)	11.5 (2.46)	11.6 (2.56)
**Days of one menstrual cycle**	29.5 (1.12)	29.4 (1.18)	29.6 (1.20)	29.6 (1.23)
**Years of dysmenorrhea history**	9.2 (2.70)	9.3 (2.79)		
**Days of menstrual pain per cycle**	1.8 (0.82)	1.9 (0.83)		
**MPQ: recalled pain rating index**	33.3 (12.96)	34.5 (13.17)		
**MPQ: recalled pain intensity**	3.1 (1.21)	3.1 (1.17)		

Abbreviations: MPQ, McGill Pain Questionnaire; PDM, primary dysmenorrhea. The data are presented as the means (SD).

Of all the PDMs and controls who received genetic testing, 21 PDMs were excluded from the genetic-behavioral-hormonal-multimodal neuroimaging studies for the following reasons: appendicitis after recruitment (n = 1), fifth ventricle in the brain (n = 2), arteriovenous malformation in the brain (n = 1), MRI claustrophobia (n = 2), irregular menstrual cycle after recheck (n = 1), and withdrawal with incomplete behavioral/hormonal data (n = 14). Twenty controls were excluded from the genetic-behavioral-hormonal-multimodal neuroimaging studies for the following reasons: intracranial arachnoid cyst (n = 1), irregular menstrual cycle after recheck (n = 1), development of systemic lupus erythematosus after recruitment with anti-lupus medications (n = 1), undisclosed prior head injury (n = 1), and withdrawal with incomplete data (n = 16). The participants with the aforementioned problems and incidental MRI brain findings were excluded from further genetic-behavioral-hormonal and multimodal neuroimaging analyses. The demographic data of the remaining participants (PDM = 78, control = 81) retained similar features of the original samples (*P*>0.05).

### Genotypes and Allele Distributions (Table 2)

Participants in the two groups were all successfully genotyped. The genotype distributions of the *BDNF* Val66Met (rs6265) for both groups were in Hardy-Weinberg equilibrium (*P*>0.05; Table 2). A sample size of 200 (99 PDMs, 101 controls) achieved 64% statistical power to detect an effect size of 0.184 using a 2-degree-of-freedom chi-square test with a significance level of 0.05. The PDM group differed significantly from the control group in *BDNF* genotypes (*P* = 0.034) and alleles (*P* = 0.013) distributions. There was a significant excess of the Met allele in the PDM group compared with the control group (Table 2). The PDM prevalence difference in all *BDNF* Met/Met homozygotes (63.8%) vs. all Val carriers (43.7%) attained high significance (*P* = 0.012). Treating the *BDNF* Val allele carriers as the baseline, the odds ratio for *BDNF* Met/Met homozygosity was 2.27 (95% confidence interval = 1.21–4.27) ((PDM-Met/Met × control-Val-carriers)/(PDM-Val-carriers × control-Met/Met)). It implied that those carrying the *BDNF* Met/Met genotype had an increased risk of PDM. The *BDNF* Val66Met polymorphism had no association with the age of onset (Val carriers, 14.2±1.76 years of age; Met homozygotes, 13.9±1.56) or duration of menstrual pain per cycle (Val carriers, 1.8±0.86 days; Met/Met homozygotes, 1.8±0.75) in the PDM group.

**Table 2:** *BDNF* rs6265 genotype distributions and allele frequency.

	Genotype (n, %)				Allele frequency		
	G/G (Val/Val)	G/A (Val/Met)	A/A (Met/Met)	*P*	Val	Met	*P*
**PDM (n = 99)**	24, 24.2%	38, 38.4%	37, 37.4%	0.034	43.4%	56.6%	0.013
**Control (n = 101)**	33, 32.7%	47, 46.5%	21, 20.8%		55.9%	44.1%	

Abbreviations: *BDNF*, brain-derived neurotrophic factor; PDM, primary dysmenorrhea; A, adenine; G, guanine; Val, Valine; Met, Methionine.

For the behavioral/hormonal assessments (PDM = 78, control = 81; eligible for multimodal neuroimaging studies), the sub-groups retained the original group features in terms of the genotype and allele distributions (Table S1). No differences in demographic data were noted between the *BDNF* Met/Met homozygotes and the Val carriers in the sub-groups (all *P*>0.05).

### Differences in Behavioral Assessments

#### Menstrual pain experience (Table 3)

During the menstrual phase, differences in pain rating index (*P* = 0.131) and present pain intensity (*P* = 0.925) of McGill Pain Questionnaire were not demonstrated between the *BDNF* Met/Met homozygous PDMs and the Val carrier PDMs. However, the *BDNF* Met/Met homozygous PDMs scored significantly higher on the sensory subscale of McGill Pain Questionnaire compared with the Val carriers (*P* = 0.017).

**Table 3:** *BDNF* rs6265 genotype effect on McGill Pain Questionnaire scores of PDMs during the menstrual phase.

		Met/Met	Val carrier	*P*
**Subject number**		29	49	
**Pain rating index**		32.3 (13.47)	26.7 (12.02)	0.131
** Sensory**		18.5 (6.21)	14.7 (6.13)	0.017
** Affective**		4.2 (3.57)	3.4 (2.29)	0.554
** Evaluative**		2.4 (2.02)	2.1 (2.01)	0.613
** Miscellaneous**		7.2 (3.72)	6.5 (3.81)	0.418
**Present pain intensity**		2.7 (1.02)	2.6 (1.01)	0.925

Abbreviations: *BDNF*, brain-derived neurotrophic factor; PDM, primary dysmenorrhea; Val, valine; Met, methionine. The data are presented as the means (SD).

#### Personality traits and quality of life (Table 4)

There was a significant main effect of group (but no significant main effect of *BDNF* genotype or interaction between group and *BDNF* genotype) on the anxiety (*F* = 10.975, *R^2^* = 0.068, *P* = 0.001), depression (*F* = 8.875, *R^2^* = 0.056, *P* = 0.003), and hypochondriasis (*F* = 27.314, *R^2^* = 0.154, *P*<0.001) dimensions of Basic Personality Inventory. Significant group effect was noted also on physical (*F* = 31.554, *R^2^* = 0.173, *P*<0.001) and mental (*F* = 40.455, *R^2^* = 0.211, *P*<0.001) components of SF-36. Regardless of genotype, the PDMs (compared with the controls) scored significantly higher on the anxiety, depression, and hypochondriasis dimensions of Basic Personality Inventory and significantly lower on the physical and mental components of SF-36.

#### Anxiety, depressive mood and pain catastrophizing (Table 5)

For the analyses of the entire cohort, there was a significant main effect of group on the state anxiety (*F* = 27.089, *R^2^* = 0.152, *P*<0.001), trait anxiety (*F* = 17.593, *R^2^* = 0.106, *P*<0.001), Beck Anxiety Inventory (*F* = 59.864, *R^2^* = 0.284, *P*<0.001), Beck Depression Inventory (*F* = 17.810, *R^2^* = 0.106, *P*<0.001), and Pain Catastrophizing Scale (*F* = 72.171, *R^2^* = 0.334, *P*<0.001) scores, with a significant interaction between menstrual cycle and group on the state anxiety (*F* = 24.269, *R^2^* = 0.138, *P*<0.001), Beck Anxiety Inventory (*F* = 70.943, *R^2^* = 0.320, *P*<0.001), Beck Depression Inventory (*F* = 32.477, *R^2^* = 0.177, *P*<0.001), and Pain Catastrophizing Scale (*F* = 4.412, *R^2^* = 0.030, *P* = 0.037) scores. There was also a significant main effect of menstrual cycle on the state anxiety (*F* = 16.459, *R^2^* = 0.098, *P*<0.001), Beck Anxiety Inventory (*F* = 60.690, *R^2^* = 0.287, *P*<0.001), Beck Depression Inventory (*F* = 28.713, *R^2^* = 0.160, *P*<0.001), and Pain Catastrophizing Scale (*F* = 4.514, *R^2^* = 0.030, *P* = 0.035) scores and a significant main effect of *BDNF* genotype on the state anxiety (*F* = 6.207, *R^2^* = 0.039, *P* = 0.014) and Beck Anxiety Inventory (*F* = 4.974, *R^2^* = 0.032, *P* = 0.027) scores. Significant interactions between menstrual cycle and *BDNF* genotype (*F* = 7.595, *R^2^* = 0.048, *P* = 0.007), as well as among menstrual cycle, group and *BDNF* genotype (*F* = 4.509, *R^2^* = 0.029, *P* = 0.035), on the Beck Anxiety Inventory score were noted.

In the individual group analyses, the PDM group exhibited a significant main effect of menstrual cycle on state anxiety (*F* = 33.144, *R^2^* = 0.309, *P*<0.001), Beck Anxiety Inventory (*F* = 85.498, *R^2^* = 0.536, *P*<0.001), Beck Depression Inventory (*F* = 45.579, *R^2^* = 0.381, *P*<0.001), and Pain Catastrophizing Scale (*F* = 7.556, *R^2^* = 0.095, *P* = 0.008) scores, as well as a significant interaction between menstrual cycle and *BDNF* genotype on the Beck Anxiety Inventory score (*F* = 7.743, *R^2^* = 0.095, *P* = 0.007) (Table 5). In view of *R^2^* statistics, the significant interaction between menstrual cycle and *BDNF* genotype should mainly be attributed to the main effect of menstrual cycle in the PDM group. By contrast, the control group exhibited neither significant main effects nor interactions (Table S2). Compared with the controls, the PDMs reported significantly higher state anxiety, trait anxiety, Beck Anxiety Inventory, Beck Depression Inventory, and Pain Catastrophizing Scale scores during either the menstrual phase or periovulatory phase. The PDMs had significantly higher state anxiety, Beck Anxiety Inventory, Beck Depression Inventory, and Pain Catastrophizing Scale scores, but not trait anxiety score, during the menstrual phase than the periovulatory phase. The controls demonstrated no significant between-phase differences in any of the psychological measurements. The Met/Met homozygous PDMs, compared with the Val carrier PDMs, manifested higher Beck Anxiety Inventory scores during the menstrual phase, but not during the periovulatory phase. In the controls, an absence of between-genotype differences was noted in all the psychological measurements.

#### Quantitative sensory testing results (Table S3)

Because there was no right-left difference in any of the measured thresholds in each subject (all *P*>0.05), we averaged the bilateral values for the corresponding dermatome areas for the group comparisons. No main effect of menstrual cycle, group, *BDNF* genotype or interactions between them was noted for any of the measured thresholds. In brief, we detected no regional or generalized central sensitization of superficial (skin) pain in the Taiwanese (Asian) participants.

### Serum Hormone Levels (Table S4)

There was a significant main effect of menstrual cycle, but no main effect of group or *BDNF* genotype, on the serum estradiol (*F* = 85.604, *R^2^* = 0.393, *P*<0.001), progesterone (*F* = 8.937, *R^2^* = 0.063, *P* = 0.003), and testosterone (*F* = 31.150, *R^2^* = 0.190, *P*<0.001) levels. The serum gonadal hormone levels were significantly higher during the periovulatory phase than during the menstrual phase, regardless of group or *BDNF* genotype.

## Discussion

### The Association of *BDNF* Val66Met Polymorphism with PDM

To our knowledge, this is the first study to report a significant association between the *BDNF* Val66Met polymorphism and PDM. The genotype distributions and allele frequencies of the *BDNF* Val66Met polymorphism exhibit substantial ethnic differences, which may consequently affect the results of genetic association studies. The Met allele frequency in Asian populations (40–50%) is significantly higher than that of white populations (25–32%) [15], [46]. The *BDNF* genotype distribution and allele frequency of the overall sample (Met allele frequency: 50.25%) in our study concur with previous reports on Asian populations [47], [48]. The finding that Taiwanese PDMs exhibit a significantly higher *BDNF* Met/Met prevalence and *BDNF* Met allele frequency implies that young *BDNF* Met/Met homozygous Taiwanese females may have an increased risk of PDM (Table 2).

### The Role of *BDNF* Val66Met Polymorphism as a Regulator of Menstrual Pain

In animal studies, BDNF functions either as a pro-nociceptive or an anti-nociceptive role in the brainstem pain modulatory system (midbrain periaqueductal gray and rostral ventromedial medulla). On one hand, BDNF mRNA and protein levels are upregulated in the dorsal root ganglion, spinal cord and brainstem pain modulatory system under peripheral noxious stimulation. BDNF herein exerts pro-nociceptive effects and contributes to descending pain facilitation and chronic pain hypersensitivity [49], [50]. On the other hand, BDNF can manifest protective anti-nociceptive effects under repeated painful conditions as a result of multiple mechanisms, including a direct neurotransmitter-like effect or processes of neural plasticity in the anti-nociceptive systems, induction of serotonin-related analgesia, and a modulatory effect at the supraspinal level [51]. BDNF can therefore be consociated with both an enhancement of nociceptive impact, and a dampened response to protect the brain from pain overstimulation [51]. Thus, the overall effect of BDNF on pain perception and experience depends on the net interaction output of these apparently contrasting activities at different neural structures in which BDNF is produced, secreted, and left to act [17], [50], [51]. The *BDNF* Val66Met polymorphism can lead to impaired BDNF signaling in the brainstem pain modulatory system and it may be associated with altered menstrual pain modulation in PDMs. The aforementioned dual effects of BDNF on pain may explain in part the diversity of the results of behavioral and psychological assessments, as demonstrated that *BDNF* genotype expressed an effect on the sensory dimension scores but not on the other dimension scores and the total scores (pain rating index) of McGill Pain Questionnaire reported during the menstrual phase.

PDM is not only associated with irritable bowel syndrome and fibromyalgia but is also viewed as a harbinger of these conditions [52], [53]. Central sensitization-induced viscero-visceral [44] and viscero-somatic hyperalgesia [40] outside the uterine referral area during early life can be a mechanism contributing to these chronic pain conditions later in life. In PDMs with comorbid irritable bowel syndrome, treating menstrual pain helps to relieve the pain associated with irritable bowel syndrome [54]. A role for BDNF protein in chronic pain conditions such as irritable bowel syndrome [55] and fibromyalgia [56]–[58] has been suggested. It has been postulated that BDNF protein strengthens the synaptic plasticity within the pain modulatory circuitry and contributes to descending pain facilitation and the subsequent visceral pain and hypersensitivity [50]. It has been reported that irritable bowel syndrome was associated with the up-regulation of BDNF in the intestinal mucosa and that colonic BDNF expression correlated with the severity of abdominal pain [55]. Fibromyalgia has been reported to be associated with the up-regulation of BDNF in the cerebrospinal fluid [56], and the extent of this up-regulation was correlated with the duration of chronic pain. However, it remains to be explored whether *BDNF* Val66Met polymorphism substantially modulates the process of central sensitization and whether a specific genotype(s) renders PDMs more vulnerable to the development of irritable bowel syndrome and fibromyalgia later in life.

### Personality Traits and Quality of Life of PDMs

Compared with the controls, the PDMs manifested significantly higher levels of depressive mood, anxiety, and hypochondriasis as their personality traits (Table 4). These findings are in line with previous studies [59], [60]. However, these levels did not reach the degree of clinical severity, such as an anxiety disorder, that is mandatory for psychiatric intervention. The PDMs reported a significantly lower quality of life, encompassing both physical and mental components, compared with the controls (Table 4). The absence of main effects of genotype or the interaction between group and genotype in both the Basic Personality Inventory and SF-36 assessments connotes that the *BDNF* Val66Met polymorphism does not directly influence personality traits and quality of life. Rather, menstrual pain per se is the dominant factor influencing these two life facets. Such dysmenorrhea-associated dysfunction, in its severe and protracted form, may through chronification eventually impact women’s long-term health as profoundly as chronic diseases [12].

**Table 4:** ANOVA results of Basic Personality Inventory and SF-36 assessments, stratified by group and *BDNF* genotype.

	PDM		Control		Main effect		Interaction
	Met/Met	Val carrier	Met/Met	Val carrier	Group *(P*)	Genotype *(P*)	Group*Genotype *(P*)
**Subject number**	29	49	17	64			
**Basic Personality Inventory**							
** Depression**	3.2 (2.72)	3.7 (3.44)	2.4 (2.47)	1.7 (1.87)	0.003	0.887	0.240
** Anxiety**	6.1 (3.25)	4.9 (3.42)	3.4 (3.10)	3.9 (2.70)	0.001	0.480	0.135
** Social introversion**	3.5 (2.49)	3.4 (2.86)	4.1 (2.28)	3.7 (2.57)	0.357	0.688	0.776
** Self depreciation**	2.1 (2.14)	2.5 (2.70)	2.1 (1.64)	2.6 (2.40)	0.929	0.283	0.835
** Interpersonal problems**	4.8 (2.43)	4.2 (2.22)	4.5 (3.06)	3.5 (2.71)	0.269	0.082	0.709
** Impulse expression**	4.6 (3.29)	4.4 (3.03)	4.2 (3.55)	4.5 (2.97)	0.878	0.945	0.681
** Deviation**	2.4 (1.93)	2.5 (2.34)	2.3 (1.49)	2.1 (1.95)	0.393	0.830	0.653
** Hypochondriasis**	4.6 (3.25)	4.7 (2.84)	2.4 (1.41)	2.2 (1.92)	<0.001	0.986	0.757
** Persecutory ideas**	2.7 (2.11)	2.8 (2.03)	2.8 (1.74)	2.0 (2.15)	0.393	0.342	0.205
** Thinking disorder**	1.8 (1.94)	1.9 (1.63)	1.6 (1.62)	1.5 (1.80)	0.363	0.926	0.837
**SF-36**							
** Physical component**	46.3 (10.10)	45.2 (10.12)	52.6 (5.64)	54.7 (4.08)	<0.001	0.727	0.243
** Mental component**	46.2 (7.51)	46.5 (7.19)	53.1 (4.95)	53.7 (4.69)	<0.001	0.668	0.915

Abbreviations: ANOVA, analysis of variance; *BDNF*, brain-derived neurotrophic factor; SF-36, 36-Item Short-Form Health Survey; PDM, primary dysmenorrhea; Val, valine; Met, methionine. The data are presented as the means (SD).

**Table 5:** Results of repeated-measures ANOVA of psychological measurements: effects of menstrual cycle and *BDNF* genotype in the PDM group.

	Met/Met	Val carrier	Main effect		Interaction
			Phase (*P*)	Genotype (*P*)	Phase*Genotype (*P*)
**Subject number**	29	49			
**State anxiety**					
** MENS**	45.5 (9.68)	41.1 (8.61)	<0.001	0.052	0.495
** POV**	38.9 (7.84)	36.0 (5.60)			
**Trait anxiety**					
** MENS**	45.6 (10.10)	44.0 (8.21)	0.056	0.386	0.886
** POV**	44.3 (9.37)	42.4 (8.55)			
**Beck anxiety**					
** MENS**	14.9 (6.95)	10.9 (6.82)	<0.001	0.138	0.007
** POV**	6.3 (5.96)	6.3 (5.78)			
**Beck depression**					
** MENS**	13.8 (10.52)	11.3 (9.73)	<0.001	0.429	0.295
** POV**	6.1 (7.27)	5.7 (6.94)			
**Pain catastrophizing**					
** MENS**	22.6 (11.05)	19.6 (12.58)	0.008	0.383	0.512
** POV**	18.8 (9.27)	17.3 (12.35)			

Abbreviations: ANOVA, analysis of variance;

*BDNF*, brain-derived neurotrophic factor;

PDM, primary dysmenorrhea; MENS, menstrual phase;

POV, periovulatory phase;

Val, valine; Met, methionine.

The data are presented as the means (SD).

### The Possible Role of *BDNF* Val66Met Polymorphism as a Regulator of Emotions in PDM

The PDMs exhibited more prominent negative moods (anxiety and depression) and pain catastrophizing compared with the controls (Table 5 and Table S2). Women with high pain catastrophizing scores may have altered cognitive-affective processing of menstrual pain-laden emotional orientations, which may precipitate depression, anxiety and altered pain experience [61]. Although the interaction between the *BDNF* genotype and menstrual cycle for Beck Anxiety Inventory scores in the PDM group was not substantial, we found an effect of the gene (*BDNF* genotype) × environment (menstrual pain) interaction on the Beck Anxiety Inventory scores when considering menstrual pain as an environmental stressor. Additionally, the *BDNF* Met/Met homozygous PDMs displayed higher anxiety during the menstrual phase compared with the Val carrier PDMs (Table 5). It is noteworthy that the *BDNF* Val66Met polymorphism produced no significant effects on mood across the menstrual cycle in the control group. These findings collectively connote that the *BDNF* Val66Met polymorphism is modestly associated with the supraspinal modulation of menstrual pain-laden emotional processing. Although *BDNF* Val66Met polymorphism alone may not effectuate depressive and anxious moods, it may represent a predisposing factor, and chronic stress like cyclic menstrual pain is necessary for the detection [62]. Our arguments are in agreement with a meta-analysis that concluded that *BDNF* Met/Met homozygous Asians are predisposed to a sub-significantly higher risk of anxiety disorders [28].

The possible mechanisms underlying the modulatory function of the *BDNF* Val66Met polymorphism on mood may be manifold. According to the neurotrophic model for stress-related mood disorders [19], Met/Met homozygotes may exhibit a deficient expression of BDNF in the mood control areas of the brain during stress (e.g., chronic pain [20]), which may predispose vulnerable subjects to mood disorders [63]. This hypothesis is corroborated by animal studies in which Met/Met homozygotes exhibited defective activity-dependent BDNF secretion from neurons and manifested anxiety-related behaviors in stressful settings [25]. Furthermore, declines in estrogen (as a positive regulator that enhances BDNF expression) during the menstrual phase may lead to a decreased expression of BDNF in the brain, which in turn could hamper the BDNF signaling in the mood-controlling networks of susceptible subjects [21], [22]. This study did not reveal a group or genotype main effect (or the interaction between them) on gonadal hormones (Table S4). Thus, the role of estrogen as a regulator of PDM-related emotions remains elusive.

### Absence of Central Sensitization to Experimental Skin Pain

Having a sample size many times greater than those reported in the literature [40]–[43], we found no group (PDM vs. control), menstrual cycle (menstrual phase vs. periovulatory phase), or genotype (*BDNF* Met/Met homozygotes vs. Val carriers) effects on pain sensitivity to experimental skin thermal stimuli (Table S3). Our findings are at odds with published studies (based on very small samples of 10–22 PDMs and 10–31 controls; most studies had 10 of each) that dysmenorrheic white women, compared with non-dysmenorrheic ones, exhibit heightened pain sensitivity or central sensitization (either regional or generalized) to experimental somatic [40]–[43] and visceral stimuli [44]. Notwithstanding, other studies on dysmenorrheic white women have also yielded conflicting results [43], [64]. Our results indicate that additional factors contributing to the diversity of the clinical manifestations of PDM may include different genetic constitutions (in the context of ethnicity) and the diverse nature and quality of experimental pain caused by different stimuli [65]. Future studies employing imaging genetics [66] in combination with more comprehensively designed psychophysical experiments (including deep somatic pain and visceral pain assessments) on a larger sample may be helpful to elucidate the supraspinal (brain) mechanisms that underlie the presence or absence of central sensitization in the context of ethnic differences. It should be born in mind that such mechanisms of presence or absence of central sensitization may occur at different level of the neuraxis of the central nervous system.

### Limitations and Further Considerations

Our study indicates an association of the *BDNF* Val66Met polymorphism with PDM, and our data suggest, yet not strongly affirm, a possible role of *BDNF* Val66Met polymorphism as a regulator of menstrual pain and pain-related emotions in PDM. The discordance between genetic association and clinical manifestations in our study has been corroborated by a previous study on Taiwanese that reported the genetic association of the *BDNF* Val66Met polymorphism with a higher risk of geriatric depression, but not with the disease severity (depression and cognitive deficit) [30]. Some possible explanations are suggested below. Firstly, we speculate that the overall manifestations of pain experience of PDM may be determined in part by the net output of contrasting pro- and anti-nociceptive effect of BDNF in the pain circuitry, that may result in the discordance between the genetic risk predisposition and pain-emotional expressions. This can be better appreciated in the context that PDM, dissimilar with major affective disorders and neurological diseases, usually does not manifest intense mood liability and functional disability except the severe form that leads to absenteeism from school and work. Secondly, the *BDNF* Val66Met polymorphism, as an individual gene, may not play a dominant role in the overall expressions of menstrual pain and pain-related emotions, in which polygenic interactions and regulations (e.g., genes related to the serotonergic and dopaminergic systems) are most likely involved [67]–[69]. Thirdly, the behavioral assessments we used may not perfectly reflect the pain- and emotion-related experiential facets of PDM. Fourthly, functional variants within the coding or regulatory regions of the *BDNF*, other than the *BDNF* Val66Met polymorphism, might also contribute to the clinical manifestations of PDM. The *BDNF* G-712A polymorphism, a genetic variant located in the putative promoter region [70], has been reported to be associated with major depression in Chinese [71]. Lastly, the *BDNF* Val66Met polymorphism may not be directly involved in the pathogenesis of PDM, and the association between the *BDNF* Val66Met polymorphism and PDM may be through linkage disequilibrium with other genetic variants of the *BDNF* or nearby genes.

The sample sizes in the current report are relatively small due to the rigorous inclusion/exclusion criteria and the demanding processes of associated longitudinal multimodal neuroimaging studies for each participant. We consider the novel findings reported in this study as preliminary. However, our observations do echo the recent mandate for a thorough revisit of PDM [4], [52], and invite further studies of larger sample size for the genetic and neuroscientific underpinnings of the clinical (pain and related emotions) and psychophysical (pain sensitivity) manifestations of PDM females.

## Conclusions

Our data suggest that the *BDNF* Val66Met polymorphism is associated with PDM in Taiwanese (Asian) people. *BDNF* Met/Met homozygous young Taiwanese females may have an increased risk of PDM. The putative role of *BDNF* Val66Met polymorphism as a supraspinal regulator of menstrual pain and pain-laden emotions remains to be further verified. Absence of thermal hypersensitivity may connote an ethnic attribution. Ethnicity of different genetic constitutions should be taken into consideration in future PDM research.

## Supporting Information

Table S1 ***BDNF*** **rs6265 genotype distributions and allele frequency in the behavioral/hormonal sub-groups.**
(DOC)

Table S2 **Results of repeated-measures ANOVA of psychological measurements: effects of menstrual cycle and** ***BDNF*** **genotype in the control group.**
(DOC)

Table S3 **Results of repeated-measures ANOVA of quantitative sensory testing: effects of group,** ***BDNF*** **genotype and menstrual cycle.**
(DOC)

Table S4 **Results of repeated-measures ANOVA of gonadal hormone levels: effects of group,** ***BDNF*** **genotype and menstrual cycle.**
(DOC)

## References

[1] French L (2005) Dysmenorrhea. Am Fam Physician 71: 285–291. 15686299

[2] Davis AR, Westhoff CL (2001) Primary dysmenorrhea in adolescent girls and treatment with oral contraceptives. J Pediatr Adolesc Gynecol 14: 3–8. 11358700 10.1016/s1083-3188(00)00076-0

[3] Fedorowicz Z, Nasser M, Jagannath VA, Beaman JH, Ejaz K, et al (2012) Beta2-adrenoceptor agonists for dysmenorrhoea. Cochrane Database Syst Rev 5: Cd008585. 10.1002/14651858.CD008585.pub2 PMC11365590 22592732

[4] Berkley KJ (2013) Primary dysmenorrhea: an urgent mandate. Pain: Clinical Updates XXI NO3: 1–8.

[5] Guo SW, Mao X, Ma Q, Liu X (2013) Dysmenorrhea and its severity are associated with increased uterine contractility and overexpression of oxytocin receptor (OTR) in women with symptomatic adenomyosis. Fertil Steril 99: 231–240. 22999795 10.1016/j.fertnstert.2012.08.038

[6] Akerlund M (1994) Vascularization of human endometrium. Uterine blood flow in healthy condition and in primary dysmenorrhoea. Ann N Y Acad Sci 734: 47–56. 7978951 10.1111/j.1749-6632.1994.tb21735.x

[7] Ma H, Hong M, Duan J, Liu P, Fan X, et al (2013) Altered cytokine gene expression in peripheral blood monocytes across the menstrual cycle in primary dysmenorrhea: a case-control study. PLoS One 8: e55200. 23390521 10.1371/journal.pone.0055200 PMC3563666

[8] Abu JI, Konje JC (2000) Leukotrienes in gynaecology: the hypothetical value of anti-leukotriene therapy in dysmenorrhoea and endometriosis. Hum Reprod Update 6: 200–205. 10782578 10.1093/humupd/6.2.200

[9] Akerlund M (2006) Targeting the oxytocin receptor to relax the myometrium. Expert Opin Ther Targets 10: 423–427. 16706682 10.1517/14728222.10.3.423

[10] Tzafettas J (2006) Painful menstruation. Pediatr Endocrinol Rev 3 Suppl 1: 160–163. 16641851

[11] Sundell G, Milsom I, Andersch B (1990) Factors influencing the prevalence and severity of dysmenorrhoea in young women. Br J Obstet Gynaecol 97: 588–594. 2390501 10.1111/j.1471-0528.1990.tb02545.x

[12] Barnard K, Frayne SM, Skinner KM, Sullivan LM (2003) Health status among women with menstrual symptoms. J Womens Health (Larchmt) 12: 911–919. 14670171 10.1089/154099903770948140

[13] Alonso C, Coe CL (2001) Disruptions of social relationships accentuate the association between emotional distress and menstrual pain in young women. Health Psychol 20: 411–416. 11714182

[14] Dorn LD, Negriff S, Huang B, Pabst S, Hillman J, et al (2009) Menstrual symptoms in adolescent girls: association with smoking, depressive symptoms, and anxiety. J Adolesc Health 44: 237–243. 19237109 10.1016/j.jadohealth.2008.07.018 PMC2667342

[15] Verhagen M, van der Meij A, van Deurzen PA, Janzing JG, Arias-Vasquez A, et al (2010) Meta-analysis of the BDNF Val66Met polymorphism in major depressive disorder: effects of gender and ethnicity. Mol Psychiatry 15: 260–271. 18852698 10.1038/mp.2008.109

[16] Lommatzsch M, Zingler D, Schuhbaeck K, Schloetcke K, Zingler C, et al (2005) The impact of age, weight and gender on BDNF levels in human platelets and plasma. Neurobiol Aging 26: 115–123. 15585351 10.1016/j.neurobiolaging.2004.03.002

[17] Merighi A, Salio C, Ghirri A, Lossi L, Ferrini F, et al (2008) BDNF as a pain modulator. Prog Neurobiol 85: 297–317. 18514997 10.1016/j.pneurobio.2008.04.004

[18] Latremoliere A, Woolf CJ (2009) Central sensitization: a generator of pain hypersensitivity by central neural plasticity. J Pain 10: 895–926. 19712899 10.1016/j.jpain.2009.06.012 PMC2750819

[19] Duman RS, Monteggia LM (2006) A neurotrophic model for stress-related mood disorders. Biol Psychiatry 59: 1116–1127. 16631126 10.1016/j.biopsych.2006.02.013

[20] Duric V, McCarson KE (2006) Persistent pain produces stress-like alterations in hippocampal neurogenesis and gene expression. J Pain 7: 544–555. 16885011 10.1016/j.jpain.2006.01.458

[21] Spencer JL, Waters EM, Milner TA, Lee FS, McEwen BS (2010) BDNF variant Val66Met interacts with estrous cycle in the control of hippocampal function. Proc Natl Acad Sci U S A 107: 4395–4400. 20142488 10.1073/pnas.0915105107 PMC2840147

[22] Bath KG, Chuang J, Spencer-Segal JL, Amso D, Altemus M, et al (2012) Variant brain-derived neurotrophic factor (Valine66Methionine) polymorphism contributes to developmental and estrous stage-specific expression of anxiety-like behavior in female mice. Biol Psychiatry 72: 499–504. 22552045 10.1016/j.biopsych.2012.03.032 PMC3414635

[23] Solum DT, Handa RJ (2002) Estrogen regulates the development of brain-derived neurotrophic factor mRNA and protein in the rat hippocampus. J Neurosci 22: 2650–2659. 11923430 10.1523/JNEUROSCI.22-07-02650.2002 PMC6758321

[24] Blurton-Jones M, Kuan PN, Tuszynski MH (2004) Anatomical evidence for transsynaptic influences of estrogen on brain-derived neurotrophic factor expression. J Comp Neurol 468: 347–360. 14681930 10.1002/cne.10989

[25] Chen ZY, Jing D, Bath KG, Ieraci A, Khan T, et al (2006) Genetic variant BDNF (Val66Met) polymorphism alters anxiety-related behavior. Science 314: 140–143. 17023662 10.1126/science.1129663 PMC1880880

[26] Vossen H, Kenis G, Rutten B, van Os J, Hermens H, et al (2010) The genetic influence on the cortical processing of experimental pain and the moderating effect of pain status. PLoS One 5: e13641. 21049025 10.1371/journal.pone.0013641 PMC2964315

[27] Yu H, Wang DD, Wang Y, Liu T, Lee FS, et al (2012) Variant brain-derived neurotrophic factor Val66Met polymorphism alters vulnerability to stress and response to antidepressants. J Neurosci 32: 4092–4101. 22442074 10.1523/JNEUROSCI.5048-11.2012 PMC3319323

[28] Frustaci A, Pozzi G, Gianfagna F, Manzoli L, Boccia S (2008) Meta-analysis of the brain-derived neurotrophic factor gene (BDNF) Val66Met polymorphism in anxiety disorders and anxiety-related personality traits. Neuropsychobiology 58: 163–170. 19088493 10.1159/000182892

[29] Montag C, Basten U, Stelzel C, Fiebach CJ, Reuter M (2010) The BDNF Val66Met polymorphism and anxiety: support for animal knock-in studies from a genetic association study in humans. Psychiatry Res 179: 86–90. 20478625 10.1016/j.psychres.2008.08.005

[30] Hwang JP, Tsai SJ, Hong CJ, Yang CH, Lirng JF, et al (2006) The Val66Met polymorphism of the brain-derived neurotrophic-factor gene is associated with geriatric depression. Neurobiol Aging 27: 1834–1837. 16343697 10.1016/j.neurobiolaging.2005.10.013

[31] Holden RR, Fekken GC, Reddon JR, Helmes E, Jackson DN (1988) Clinical reliabilities and validities of the Basic Personality Inventory. J Consult Clin Psychol 56: 766–768. 3192794 10.1037//0022-006x.56.5.766

[32] Ware J, Snow K, Kosinski M, Gandek B (1993) SF-36 Health Survey Manual and Interpretation Guide. Boston, MA: The Health Institute, New England Medical Center.

[33] Hwang RJ, Chen LF, Yeh TC, Tu PC, Tu CH, et al (2008) The resting frontal alpha asymmetry across the menstrual cycle: a magnetoencephalographic study. Horm Behav 54: 28–33. 18325518 10.1016/j.yhbeh.2007.11.007

[34] Hwang RJ, Wu CH, Chen LF, Yeh TC, Hsieh JC (2009) Female menstrual phases modulate human prefrontal asymmetry: a magnetoencephalographic study. Horm Behav 55: 203–209. 19013172 10.1016/j.yhbeh.2008.10.008

[35] Tu CH, Niddam DM, Yeh TC, Lirng JF, Cheng CM, et al (2013) Menstrual pain is associated with rapid structural alterations in the brain. Pain 154: 1718–1724. 23693160 10.1016/j.pain.2013.05.022

[36] Spielberger C, Gorsuch R, Lushene R, Vagg P, Jacobs G (1983) Manual for the State-Trait Anxiety Inventory (form Y). Palo Alto, CA: Consulting Psychologists Press.

[37] Beck A, Steer R (1993) Manual for the Beck Anxiety Inventory. San Antonio, TX: Psychological Corporation.

[38] Beck A, Steer R, Brown G (1996) Manual for Beck Depression Inventory-II. San Antonio, TX: Psychological Corporation.

[39] Sullivan MJ, Bishop S, Pivik J (1995) The pain catastrophizing scale: development and validation. Psychological Assessment 7: 524–532.

[40] Giamberardino MA, Berkley KJ, Iezzi S, de Bigontina P, Vecchiet L (1997) Pain threshold variations in somatic wall tissues as a function of menstrual cycle, segmental site and tissue depth in non-dysmenorrheic women, dysmenorrheic women and men. Pain 71: 187–197. 9211480 10.1016/s0304-3959(97)03362-9

[41] Granot M, Yarnitsky D, Itskovitz-Eldor J, Granovsky Y, Peer E, et al (2001) Pain perception in women with dysmenorrhea. Obstet Gynecol 98: 407–411. 11530120 10.1016/s0029-7844(01)01465-x

[42] Bajaj P, Bajaj P, Madsen H, Arendt-Nielsen L (2002) A comparison of modality-specific somatosensory changes during menstruation in dysmenorrheic and nondysmenorrheic women. Clin J Pain 18: 180–190. 12048420 10.1097/00002508-200205000-00007

[43] Amodei N, Nelson-Gray RO (1989) Reactions of dysmenorrheic and nondysmenorrheic women to experimentally induced pain throughout the menstrual cycle. J Behav Med 12: 373–385. 2600965 10.1007/BF00844930

[44] Brinkert W, Dimcevski G, Arendt-Nielsen L, Drewes AM, Wilder-Smith OH (2007) Dysmenorrhoea is associated with hypersensitivity in the sigmoid colon and rectum. Pain 132 Suppl 1: S46–51. 17257758 10.1016/j.pain.2006.12.011

[45] Rolke R, Magerl W, Campbell KA, Schalber C, Caspari S, et al (2006) Quantitative sensory testing: a comprehensive protocol for clinical trials. Eur J Pain 10: 77–88. 16291301 10.1016/j.ejpain.2005.02.003

[46] Petryshen TL, Sabeti PC, Aldinger KA, Fry B, Fan JB, et al (2010) Population genetic study of the brain-derived neurotrophic factor (BDNF) gene. Mol Psychiatry 15: 810–815. 19255578 10.1038/mp.2009.24 PMC2888876

[47] Hong CJ, Huo SJ, Yen FC, Tung CL, Pan GM, et al (2003) Association study of a brain-derived neurotrophic-factor genetic polymorphism and mood disorders, age of onset and suicidal behavior. Neuropsychobiology 48: 186–189. 14673216 10.1159/000074636

[48] Choi MJ, Kang RH, Lim SW, Oh KS, Lee MS (2006) Brain-derived neurotrophic factor gene polymorphism (Val66Met) and citalopram response in major depressive disorder. Brain Res 1118: 176–182. 16979146 10.1016/j.brainres.2006.08.012

[49] Obata K, Noguchi K (2006) BDNF in sensory neurons and chronic pain. Neurosci Res 55: 1–10. 16516994 10.1016/j.neures.2006.01.005

[50] Ren K, Dubner R (2007) Pain Facilitation and Activity-Dependent Plasticity in Pain Modulatory Circuitry: Role of BDNF-TrkB Signaling and NMDA Receptors. Mol Neurobiol 35: 224–235. 17917111 10.1007/s12035-007-0028-8

[51] Di Lorenzo C, Di Lorenzo G, Daverio A, Pasqualetti P, Coppola G, et al (2012) The Val66Met Polymorphism of the BDNF Gene Influences Trigeminal Pain-Related Evoked Responses. J Pain 13: 866–873. 22901763 10.1016/j.jpain.2012.05.014

[52] Berkley KJ, McAllister SL (2011) Don't dismiss dysmenorrhea! Pain. 152: 1940–1941. 10.1016/j.pain.2011.04.013 21514053

[53] Westling AM, Tu FF, Griffith JW, Hellman KM (2013) The association of dysmenorrhea with noncyclic pelvic pain accounting for psychological factors. Am J Obstet Gynecol 209: 422.e1–422.e10. 23973396 10.1016/j.ajog.2013.08.020 PMC4191839

[54] Giamberardino MA, Costantini R, Affaitati G, Fabrizio A, Lapenna D, et al (2010) Viscero-visceral hyperalgesia: characterization in different clinical models. Pain 151: 307–322. 20638177 10.1016/j.pain.2010.06.023

[55] Yu YB, Zuo XL, Zhao QJ, Chen FX, Yang J, et al (2012) Brain-derived neurotrophic factor contributes to abdominal pain in irritable bowel syndrome. Gut 61: 685–694. 21997550 10.1136/gutjnl-2011-300265

[56] Sarchielli P, Mancini ML, Floridi A, Coppola F, Rossi C, et al (2007) Increased levels of neurotrophins are not specific for chronic migraine: evidence from primary fibromyalgia syndrome. J Pain 8: 737–745. 17611164 10.1016/j.jpain.2007.05.002

[57] Haas L, Portela LV, Bohmer AE, Oses JP, Lara DR (2010) Increased plasma levels of brain derived neurotrophic factor (BDNF) in patients with fibromyalgia. Neurochem Res 35: 830–834. 20119637 10.1007/s11064-010-0129-z

[58] Nugraha B, Karst M, Engeli S, Gutenbrunner C (2012) Brain-derived neurotrophic factor and exercise in fibromyalgia syndrome patients: a mini review. Rheumatol Int 32: 2593–2599. 22210272 10.1007/s00296-011-2348-2

[59] Tu C-H, Niddam DM, Chao H-T, Liu R-S, Hwang R-J, et al (2009) Abnormal cerebral metabolism during menstrual pain in primary dysmenorrhea. Neuroimage 47: 28–35. 19362153 10.1016/j.neuroimage.2009.03.080

[60] Vincent K, Warnaby C, Stagg CJ, Moore J, Kennedy S, et al (2011) Dysmenorrhoea is associated with central changes in otherwise healthy women. Pain 152: 1966–1975. 21524851 10.1016/j.pain.2011.03.029

[61] Quartana PJ, Campbell CM, Edwards RR (2009) Pain catastrophizing: a critical review. Expert Rev Neurother 9: 745–758. 19402782 10.1586/ERN.09.34 PMC2696024

[62] Groves JO (2007) Is it time to reassess the BDNF hypothesis of depression? Mol Psychiatry 12: 1079–1088. 17700574 10.1038/sj.mp.4002075

[63] Gatt JM, Nemeroff CB, Dobson-Stone C, Paul RH, Bryant RA, et al (2009) Interactions between BDNF Val66Met polymorphism and early life stress predict brain and arousal pathways to syndromal depression and anxiety. Mol Psychiatry 14: 681–695. 19153574 10.1038/mp.2008.143

[64] Hapidou EG, De Catanzaro D (1988) Sensitivity to cold pressor pain in dysmenorrheic and non-dysmenorrheic women as a function of menstrual cycle phase. Pain 34: 277–283. 3186275 10.1016/0304-3959(88)90123-6

[65] Sherman JJ, LeResche L (2006) Does experimental pain response vary across the menstrual cycle? A methodological review. Am J Physiol Regul Integr Comp Physiol 291: R245–256. 16484434 10.1152/ajpregu.00920.2005

[66] Hariri AR, Drabant EM, Weinberger DR (2006) Imaging genetics: perspectives from studies of genetically driven variation in serotonin function and corticolimbic affective processing. Biol Psychiatry 59: 888–897. 16442081 10.1016/j.biopsych.2005.11.005

[67] Martinowich K, Lu B (2008) Interaction between BDNF and serotonin: role in mood disorders. Neuropsychopharmacology 33: 73–83. 17882234 10.1038/sj.npp.1301571

[68] Ignacio ZM, Reus GZ, Abelaira HM, Quevedo J (2014) Epigenetic and epistatic interactions between serotonin transporter and brain-derived neurotrophic factor genetic polymorphism: Insights in depression. Neuroscience 275c: 455–468. 10.1016/j.neuroscience.2014.06.036 24972302

[69] Hunnerkopf R, Strobel A, Gutknecht L, Brocke B, Lesch KP (2007) Interaction between BDNF Val66Met and dopamine transporter gene variation influences anxiety-related traits. Neuropsychopharmacology 32: 2552–2560. 17392738 10.1038/sj.npp.1301383

[70] Zhang H, Ozbay F, Lappalainen J, Kranzler HR, van Dyck CH, et al (2006) Brain derived neurotrophic factor (BDNF) gene variants and Alzheimer’s disease, affective disorders, posttraumatic stress disorder, schizophrenia, and substance dependence. Am J Med Genet B Neuropsychiatr Genet 141B: 387–393. 16649215 10.1002/ajmg.b.30332 PMC2567822

[71] Sun RF, Zhu YS, Kuang WJ, Liu Y, Li SB (2011) The G-712A polymorphism of brain-derived neurotrophic factor is associated with major depression but not schizophrenia. Neurosci Lett 489: 34–37. 21129438 10.1016/j.neulet.2010.11.061

