# Supplementary material for: Association of Brain-Derived Neurotrophic Factor Gene Val66Met Polymorphism with Primary Dysmenorrhea
Source: PLoS One. 2014 Nov 10;9(11):e112766. doi: 10.1371/journal.pone.0112766 (PMC4226574; doi:10.1371/journal.pone.0112766)
Supplement: Table S1 — BDNF rs6265 genotype distributions and allele frequency in the behavioral/hormonal sub-groups. (DOC) [file pone.0112766.s001.doc]

**Table S1.** *BDNF* rs6265 genotype distributions and allele frequency in the behavioral/hormonal sub-groups.

|  | **Genotype (n, %)** | | | | **Allele frequency** | | |
| --- | --- | --- | --- | --- | --- | --- | --- |
|  | **G/G (Val/Val)** | **G/A (Val/Met)** | **A/A (Met/Met)** | ***P*** | **Val** | **Met** | ***P*** |
| **PDM (n = 78)** | 19, 24.3% | 30, 38.5% | 29, 37.2% | 0.079 | 43.6% | 56.4% | 0.045 |
| **Control (n = 81)** | 25, 30.9% | 39, 48.1% | 17, 21.0% |  | 54.9% | 45.1% |  |

Abbreviations: *BDNF*, brain-derived neurotrophic factor; PDM, primary dysmenorrhea; A, adenine; G, guanine; Val, Valine; Met, Methionine.
