# Supplementary material for: Association of Brain-Derived Neurotrophic Factor Gene Val66Met Polymorphism with Primary Dysmenorrhea
Source: PLoS One. 2014 Nov 10;9(11):e112766. doi: 10.1371/journal.pone.0112766 (PMC4226574; doi:10.1371/journal.pone.0112766)
Supplement: Table S2 — Results of repeated-measures ANOVA of psychological measurements: effects of menstrual cycle and BDNF genotype in the control group. (DOC) [file pone.0112766.s002.doc]

**Table S2.** Results of repeated-measures ANOVA of psychological measurements: effects of menstrual cycle and *BDNF* genotype in the control group

|  |  | **Met/Met** | **Val carrier** | **Main effect** | | **Interaction** |
| --- | --- | --- | --- | --- | --- | --- |
|  |  |  |  | **Phase (*P*)** | **Genotype (*P*)** | **Phase*Genotype (*P*)** |
| **Subject number** | | 17 | 64 |  |  |  |
| **State anxiety** | |  |  |  |  |  |
|  | **MENS** | 34.8 (6.18) | 33.4 (6.55) | 0.460 | 0.218 | 0.355 |
|  | **POV** | 36.1 (6.81) | 33.3 (7.06) |  |  |  |
| **Trait anxiety** | |  |  |  |  |  |
|  | **MENS** | 38.9 (8.13) | 37.7 (7.22) | 0.885 | 0.492 | 0.895 |
|  | **POV** | 38.7 (7.53) | 37.5 (7.05) |  |  |  |
| **Beck anxiety** | |  |  |  |  |  |
|  | **MENS** | 4.2 (4.31) | 2.4 (2.24) | 0.398 | 0.055 | 0.388 |
|  | **POV** | 4.1 (3.70) | 2.9 (3.07) |  |  |  |
| **Beck depression** | |  |  |  |  |  |
|  | **MENS** | 4.7 (2.59) | 4.2 (4.36) | 0.741 | 0.268 | 0.236 |
|  | **POV** | 5.7 (5.50) | 3.7 (4.81) |  |  |  |
| **Pain catastrophizing** | |  |  |  |  |  |
|  | **MENS** | 5.4 (6.89) | 6.3 (6.69) | 0.984 | 0.645 | 0.978 |
|  | **POV** | 5.3 (5.96) | 6.2 (7.87) |  |  |  |

Abbreviations: ANOVA, analysis of variance; *BDNF*, brain-derived neurotrophic factor; MENS, menstrual phase; POV, periovulatory phase; Val, valine; Met, methionine. The data are presented as the means (SD).
