# Supplementary material for: Association of Brain-Derived Neurotrophic Factor Gene Val66Met Polymorphism with Primary Dysmenorrhea
Source: PLoS One. 2014 Nov 10;9(11):e112766. doi: 10.1371/journal.pone.0112766 (PMC4226574; doi:10.1371/journal.pone.0112766)
Supplement: Table S3 — Results of repeated-measures ANOVA of quantitative sensory testing: effects of group, BDNF genotype and menstrual cycle. (DOC) [file pone.0112766.s003.doc]

**Table S3.** Results of repeated-measures ANOVA of quantitative sensory testing: effects of group, *BDNF* genotype and menstrual cycle

|  |  | **PDM (°C)** | | **Control (°C)** | | **Main effect** | | | **Interaction** | | | |
| --- | --- | --- | --- | --- | --- | --- | --- | --- | --- | --- | --- | --- |
|  |  | **Met/Met** | **Val carrier** | **Met/Met** | **Val carrier** | **Phase (*P*)** | **Group (*P*)** | **Genotype (*P*)** | **Phase*Group (*P*)** | **Phase*Genotype (*P*)** | **Group*Genotype (*P*)** | **Phase*Group*Genotype (*P*)** |
| **Subject number** | | 29 | 49 | 17 | 64 |  |  |  |  |  |  |  |
| **Heat pain threshold - C7** | |  |  |  |  |  |  |  |  |  |  |  |
|  | **MENS** | 44.4 (3.02) | 43.8 (3.79) | 45.0 (3.04) | 44.6 (3.51) | 0.579 | 0.086 | 0.229 | 0.465 | 0.911 | 0.612 | 0.773 |
|  | **POV** | 44.2 (3.02) | 43.9 (3.11) | 45.4 (2.01) | 44.9 (3.36) |  |  |  |  |  |  |  |
| **Heat pain threshold - T11** | |  |  |  |  |  |  |  |  |  |  |  |
|  | **MENS** | 43.4 (2.96) | 43.0 (3.25) | 44.7 (2.47) | 43.4 (3.48) | 0.084 | 0.111 | 0.268 | 0.619 | 0.483 | 0.399 | 0.757 |
|  | **POV** | 43.5 (3.09) | 43.4 (3.18) | 45.0 (2.06) | 43.8 (3.29) |  |  |  |  |  |  |  |
| **Cold pain threshold - C7** | |  |  |  |  |  |  |  |  |  |  |  |
|  | **MENS** | 11.9 (10.45) | 11.7 (10.60) | 7.9 (9.69) | 10.0 (10.48) | 0.244 | 0.069 | 0.337 | 0.422 | 0.279 | 0.485 | 0.825 |
|  | **POV** | 10.9 (11.00) | 12.1 (10.50) | 5.1 (8.65) | 9.4 (10.44) |  |  |  |  |  |  |  |
| **Cold pain threshold - T11** | |  |  |  |  |  |  |  |  |  |  |  |
|  | **MENS** | 13.4 (11.75) | 13.8 (11.42) | 10.0 (11.25) | 13.4 (11.20) | 0.208 | 0.246 | 0.227 | 0.578 | 0.503 | 0.319 | 0.563 |
|  | **POV** | 12.7 (11.08) | 13.2 (10.17) | 7.3 (10.67) | 12.9 (10.53) |  |  |  |  |  |  |  |
| **Warm threshold - C7** | |  |  |  |  |  |  |  |  |  |  |  |
|  | **MENS** | 34.8 (1.49) | 35.3 (1.63) | 34.7 (1.15) | 34.9 (1.95) | 0.260 | 0.632 | 0.334 | 0.487 | 0.797 | 0.834 | 0.610 |
|  | **POV** | 35.0 (1.62) | 35.3 (1.44) | 35.0 (1.26) | 35.3 (2.12) |  |  |  |  |  |  |  |
| **Warm threshold - T11** | |  |  |  |  |  |  |  |  |  |  |  |
|  | **MENS** | 34.7 (1.36) | 35.0 (1.43) | 34.6 (0.86) | 34.7 (1.50) | 0.073 | 0.572 | 0.289 | 0.998 | 0.545 | 0.342 | 0.102 |
|  | **POV** | 34.8 (1.61) | 35.3 (1.86) | 34.9 (1.41) | 35.0 (1.70) |  |  |  |  |  |  |  |
| **Cold threshold - C7** | |  |  |  |  |  |  |  |  |  |  |  |
|  | **MENS** | 28.8 (1.62) | 28.1 (1.74) | 29.0 (1.64) | 28.6 (1.80) | 0.947 | 0.989 | 0.699 | 0.079 | 0.061 | 0.251 | 0.414 |
|  | **POV** | 29.0 (1.39) | 28.5 (1.48) | 28.4 (2.17) | 28.9 (1.40) |  |  |  |  |  |  |  |
| **Cold threshold - T11** | |  |  |  |  |  |  |  |  |  |  |  |
|  | **MENS** | 29.5 (1.65) | 29.3 (1.96) | 29.4 (1.32) | 29.3 (1.89) | 0.097 | 0.599 | 0.671 | 0.293 | 0.064 | 0.537 | 0.231 |
|  | **POV** | 29.3 (1.49) | 29.3 (1.55) | 28.6 (2.53) | 29.3 (1.51) |  |  |  |  |  |  |  |

Abbreviations: ANOVA, analysis of variance; *BDNF*, brain-derived neurotrophic factor; PDM, primary dysmenorrhea; MENS, menstrual phase; POV, periovulatory phase; Val, valine; Met, methionine. The data are presented as the means (SD).
