# Supplementary material for: Association of Brain-Derived Neurotrophic Factor Gene Val66Met Polymorphism with Primary Dysmenorrhea
Source: PLoS One. 2014 Nov 10;9(11):e112766. doi: 10.1371/journal.pone.0112766 (PMC4226574; doi:10.1371/journal.pone.0112766)
Supplement: Table S4 — Results of repeated-measures ANOVA of gonadal hormone levels: effects of group, BDNF genotype and menstrual cycle. (DOC) [file pone.0112766.s004.doc]

**Table S4.** Results of repeated-measures ANOVA of gonadal hormone levels: effects of group, *BDNF* genotype and menstrual cycle

|  |  | **PDM** | | **Control** | | **Main Effect** | | | **Interaction** | | | |
| --- | --- | --- | --- | --- | --- | --- | --- | --- | --- | --- | --- | --- |
|  |  | **Met/Met** | **Val carrier** | **Met/Met** | **Val carrier** | **Phase (*P*)** | **Group (*P*)** | **Genotype (*P*)** | **Phase*Group (*P*)** | **Phase*Genotype (*P*)** | **Group*Genotype (*P*)** | **Phase*Group*Genotype (*P*)** |
| **Subject number** | | 29 | 49 | 17 | 64 |  |  |  |  |  |  |  |
| **Estradiol (pg/mL)** | |  |  |  |  |  |  |  |  |  |  |  |
|  | **MENS** | 34.8 (15.81) | 36.7 (19.74) | 43.0 (18.53) | 44.0 (30.28) | < 0.001 | 0.683 | 0.564 | 0.269 | 0.653 | 0.905 | 0.873 |
|  | **POV** | 147.1 (114.65) | 155.5 (108.86) | 127.0 (97.53) | 141.6 (121.55) | |  |  |  |  |  |  |
| **Progesterone (ng/mL)** | |  |  |  |  |  |  |  |  |  |  |  |
|  | **MENS** | 0.5 (0.44) | 0.5 (0.39) | 0.4 (0.26) | 0.5 (0.34) | 0.003 | 0.540 | 0.213 | 0.472 | 0.308 | 0.669 | 0.643 |
|  | **POV** | 0.6 (0.82) | 1.4 (2.29) | 1.2 (2.61) | 1.5 (3.01) |  |  |  |  |  |  |  |
| **Testosterone (ng/mL)** | |  |  |  |  |  |  |  |  |  |  |  |
|  | **MENS** | 0.5 (0.23) | 0.4 (0.21) | 0.4 (0.17) | 0.4 (0.23) | < 0.001 | 0.449 | 0.081 | 0.723 | 0.061 | 0.626 | 0.074 |
|  | **POV** | 0.6 (0.36) | 0.5 (0.24) | 0.6 (0.36) | 0.4 (0.17) |  |  |  |  |  |  |  |

Abbreviations: ANOVA, analysis of variance; *BDNF*, brain-derived neurotrophic factor; PDM, primary dysmenorrhea; MENS, menstrual phase; POV, periovulatory phase; Val, valine; Met, methionine. The data are presented as the means (SD).
